# Diagnosis and Therapy of Microscopic Colitis with Presence of Foamy Macrophages in Children

**DOI:** 10.5402/2011/756292

**Published:** 2011-07-06

**Authors:** Jan Józefczuk, Bogdan Marian Woźniewicz

**Affiliations:** ^1^Pediatric Ward, Hospital in Sandomierz, Schinzla 13, 27-600 Sandomierz, Poland; ^2^Department of Pathology, The Children's Memorial Health Institute, Aleja Dzieci Polskich 20, 04-730 Warsaw, Poland

## Abstract

We discuss the diagnosis of and efficacy *5-amino-2-hydroxybenzoic acid (5-ASA), Saccharomyces boulardii, or magnesium* in therapy of microscopic colitis with presence of foamy macrophages. A basis for diagnosis and inclusion to the analysed group was presence of characteristic foamy macrophages in histopathological examination of hematoxylin and eosin-stained specimens collected from the large intestine, reviewed under ×200 or ×320 magnification. No statistically significant improvement was found following the use of *5-amino-2-dihydroxybenzoic acid* in therapy of the disease. The use of *Saccharomyces boulardii* was associated with statistically significant improvement in clinical, endoscopic, and histopathological condition. Use of magnesium caused a histological, statistically significant improvement but failed to have any effect on the clinical and endoscopic presentation. In the group of children in whom no therapeutic intervention was provided, a statistically significant spontaneous clinical improvement was observed, but no statistically significant changes in endoscopic and microscopic condition were found.

## 1. Introduction

Typical inflammatory bowel disease (IBD), such as ulcerative colitis or Crohn's disease, is only a part of cases of persistent enteritis. Nonspecific IBDs are most often superficial processes, nondamaging to the structure of intestinal mucosa and submucosa. In the medical literature, they are usually referred to as microscopic enteritis [1–4].

Foamy colitis with presence of foamy macrophages within the intestinal *lamina propria* belongs to a group of microscopic inflammations in children. That recently separated disease requires determination of effective therapeutic methods [5, 6].

Observation of that type of inflammation, indicating a rather mild clinical course of the disease and no cases of its transformation into a typical inflammatory bowel disease (IBD) justified undertaking a prospective observation of children treated with *5-amino-2-hydroxybenzoic acid*, with a probiotic (*Saccharomyces boulardii)*, or with magnesium, and of a group of children who were not treated pharmacologically.

Evaluation of clinical, endoscopic, and microscopic effects of therapy with *5-amino-2-dihydroxybenzoic acid*, *Saccharomyces boulardii*, or magnesium of children with microscopic colitis with presence of foamy macrophages and evaluation of a group of children with no therapeutic intervention.

## 2. Material and Methods

A basis for diagnosis of the disease was presence of macrophages that stained with hematoxylin and eosin formed a characteristic image of foamy (clear cell) macrophages, in the *lamina propria* of the large intestine.

Additional examinations using electron microscopy Jeol 100CX were performed in several cases. Sections of mucosa were fixed in cacodylate buffered 4% glutaraldehyde and then in osmium tetroxide (OsO_4_) and analysed acc. to a typical procedure for Epon. Resulting ultrathin sections were additionally stained with lead citrate and uranyl acetate.

Uneven distribution and quantity of foamy macrophages constituted a basis for development of *our own classification* of microscopic changes, defining three levels of intensity:

- single cells on various levels of the *lamina propria*,
- presence of aggregates composed of 3–10 cells,
- foamy cells covering evenly the whole *lamina propria*.

That type of cells was absent in other layers of the large intestine, and their presence was not found in other sections of the alimentary tract.

In the process of identification of foamy macrophages, except for hematoxylin and eosin staining of serial specimens, also a PAS reaction was used, with and without diastase digestion.

Additionally, immunocytochemical tests using monoclonal sera from DAKO: anti-CD3(T cell), CD4(T cell), CD22 (B cell), CD31 (Endothelia), CD34 (precursor cell), CD 68 (Macrophages), allowing identification of cells occurring in an inflammatory process, were completed on specimens placed on hyalinized slides. Tests were also completed to detect the PCNA-proliferation antigen, Ki67-mitotic activity, chromogranin, synaptophysin, neurofilament, actin, P53 mutations, GFAP glial marker, TNF alpha, IL2, and mucicarmin.

Tests were competed to detect intestinal pathogens (*Salmonella, Shigella, Camphylobacter, Escherichia coli, Yersnia, Clostridium difficile) *and Giardia infection.

Tests for enteroviruses were performed on cell lines (Hep 2 and RD) in the Department of Virusology of the National Health Institute, using WHO-recommended methods. Presence of rotaviruses was tested with the latex reaction, using the Slidex Rota-Kit, and completing serological tests with ELISA, using Rabbit Anti-Rotavirus Human reagents from DAKO.

Children with presence of foamy macrophages confirmed in histopathological specimens and who had a clinical and endoscopic changes characteristic for colitis were randomized to individual therapeutic groups of a prospective, open trial realised from 1999 to 2009.

In the analysis, there were included 144 children aged from 3 to 18 years. For 12 months, children received *5-amino-2-hydroxybenzoic acid (5-ASA) *at dose of 20–25 mg/kg b.w./24 h (*38 children-group A*), *Saccharomyces boulardii* at dose of 15–20 mg/kg b.w./24 h *(35 children-group B),* or *magnesium* 5–10 mg/kg b.w./24 h *(35 children-group D).*

Children who received no treatment constituted a control group (*36 children*-*group C*).

Clinical and endoscopic examinations were performed at the Pediatric Ward, and microscopic and immunocytochemical examinations—in the Department of Pathology in the Children's Memorial Health Institute in Warsaw.

Clinical evaluation included general feeling, presence of abdominal pain and its intensity, number of bowel movements per week, presence of blood in stool, fever, presence of extraenteral symptoms, ESR, haemoglobin concentration, and nutritional status expressed as Cole and Stanfield index. 

Clinical activity index (CAI) acc. to Rachmilewitz was used for evaluation of clinical presentation of analysed children [7].

The level of endoscopic lesions was determined by evaluation of vascular pattern, granulation, and mucosa proneness for bleeding, as well as presence of mucus, pus, erosions, and ulcerations on the surface of colic mucosa. The clinical activity index (CAI) acc. to Rachmilewitz was then applied [7].

Evaluation of clinical and endoscopic changes was performed at diagnosis and following 12 months of therapy and observation of each analysed child.

Due to nonparametric distribution of sets, a sign test was used for statistical evaluation of results (*Statistica *software).

## 3. Results and Discussion

**Figure 1:**
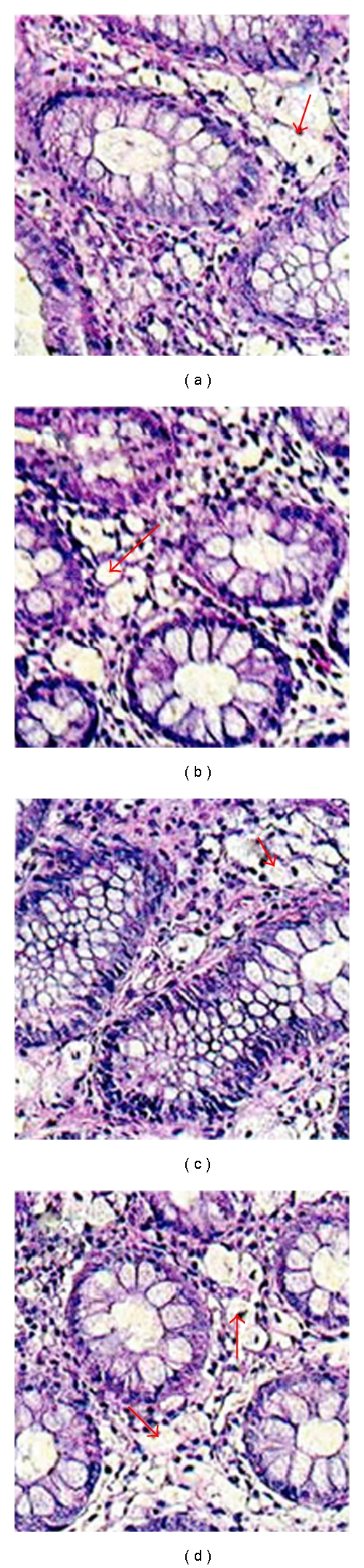
(a–d) Microscopic features of colitis with presence of foamy macrophages—characteristic large, clear cells with a small nucleus, single or aggregated within the *lamina propria. *HE staining. Magnification ×200.

At diagnosis, histopathological examination of bioptates collected from the large intestine revealed presence of large, clear, foamy cells in the *lamina propria* of the mucosa. Those cells appeared single or in agglomerates, covering the whole width of the *lamina propria—*from the covering epithelium to the base of intestinal crypts and the level of *muscularis mucosa.* (Figure 1)

**Figure 2:**
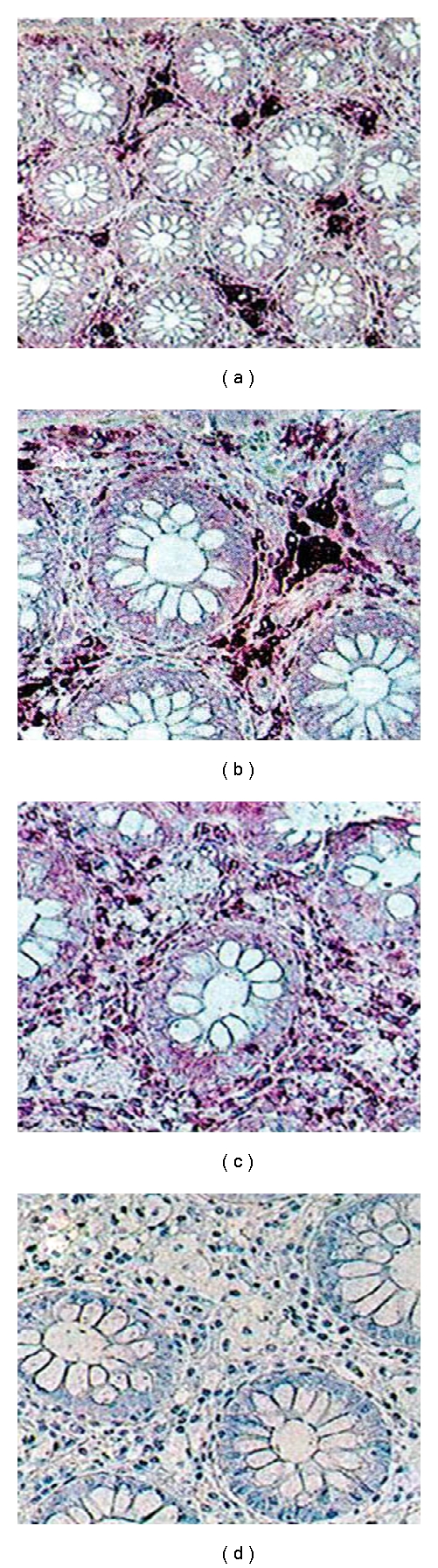
(a–d) Immunochemistry. (a-b) positive with a serum marking macrophages. C-negative to *limfocytes T*. D-neurofilament's negative reaction. Magnification ×200 (a) ×320 (b, c, d).

Immunocytochemical tests indicated a strong positive reaction with the CD68 antibody, typical for activated macrophages. Foamy macrophages showed a positive PAS reaction with periodic acid and Shiff's reagent. However, the reaction was negative following digestion with diastase. Immunocytochemical reaction to neurofilament was negative. Using the marker PCNA-proliferation antigen an increased activity was demonstrated only in cryptic epithelium. No increased activity was found in epithelium surrounding crypts (Figure 2).

Other numerous, mentioned in the Methods section, immunocytochemical markers gave a negative result in tests with foamy macrophages (Table 1).

**Figure 3:**
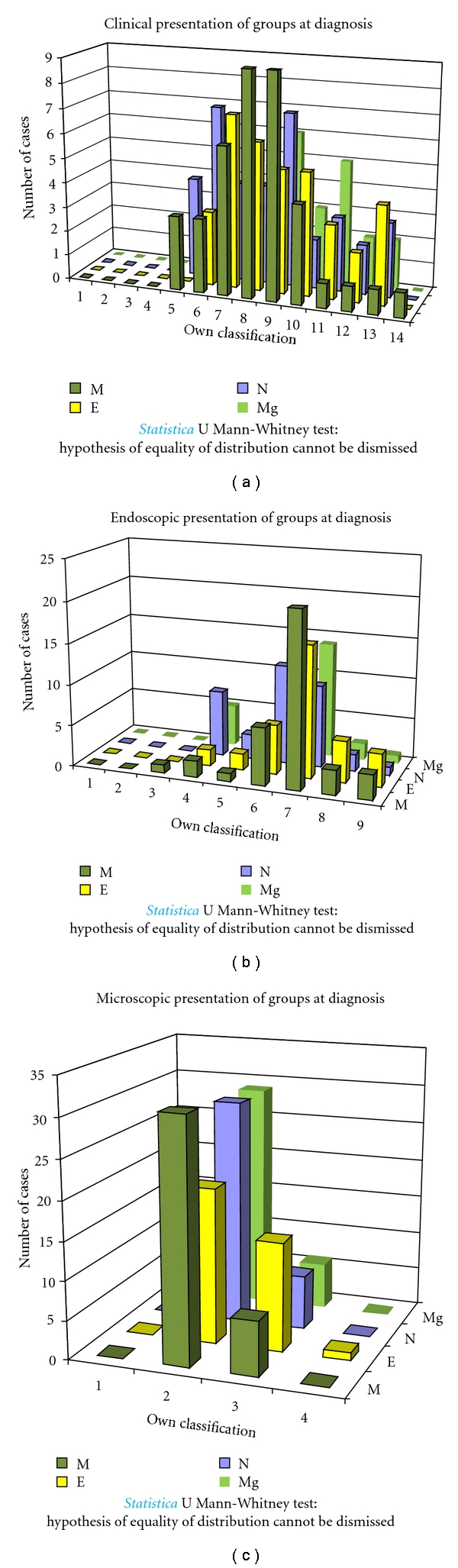
Clinical, endoscopic, and microscopic evaluation at diagnosis (Whitney-Mann test).

Before statistical analysis of results of clinical, endoscopic, and microscopic evaluation results for three study groups, they were evaluated at the time of intervention. Hypothesis of equality of those three groups (A, B, C, D) in clinical, endoscopic, and microscopic evaluation cannot be dismissed (Whitney-Mann test) (Figure 3).

**Figure 4:**
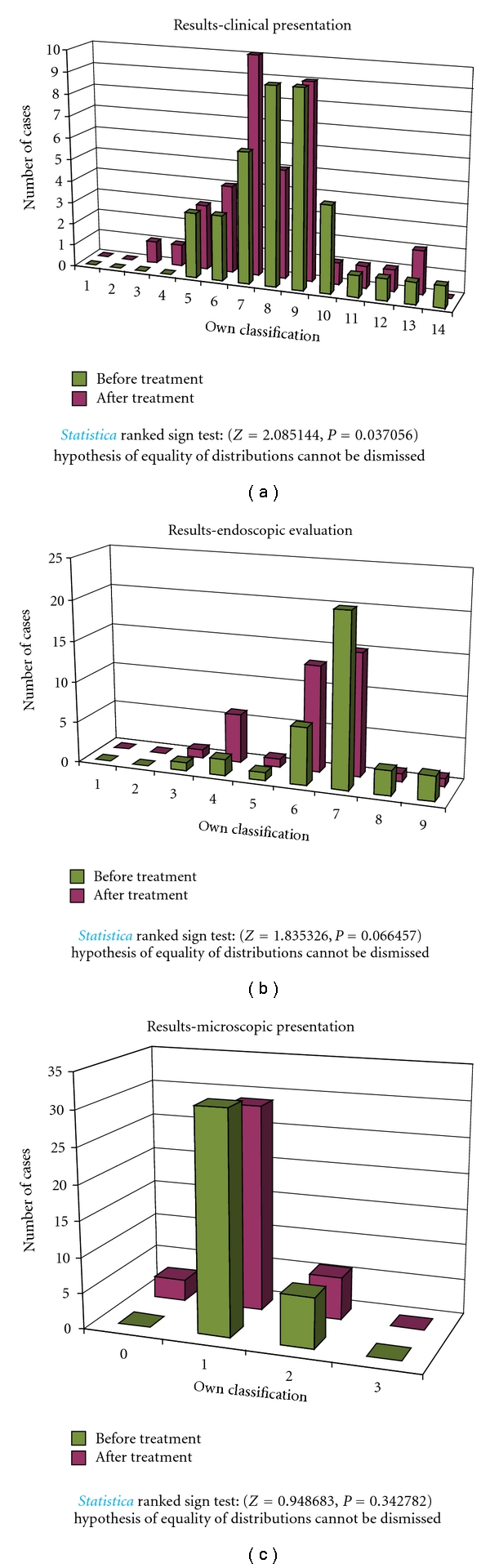
Clinical, endoscopic, and microscopic evaluation of children treated with 5-*amino-2-hydroxybenzoic acid* (group A).

In the group A, for 12 months, children received Masalazine (*5 amino-2-hydroxybenzoic acid).* Clinical, endoscopic, and microscopic evaluation of the group A, treated with *5-amino-2-hydroxybenzoic acid, *showed no significant improvement, neither in clinical, endoscopic, nor in microscopic presentation (Figure 4).

**Figure 5:**
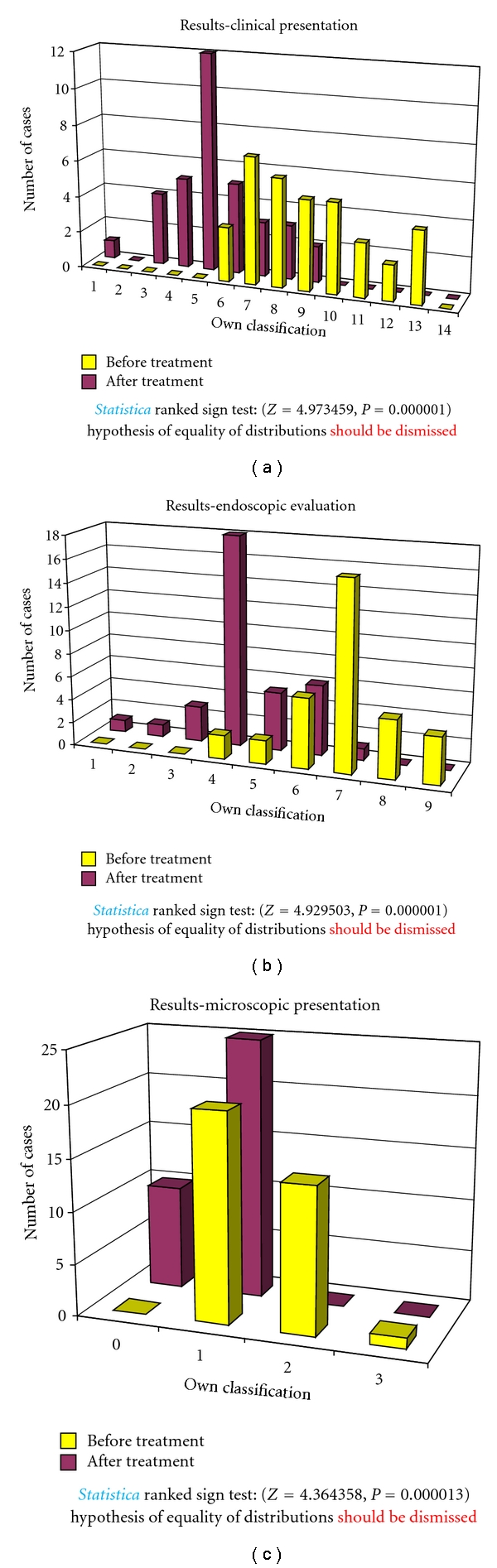
Clinical, endoscopic, and microscopic evaluation of children treated with *Saccharomyces boulardii *(group B).

In the group B, *Saccharomyces boulardii* was used for 12 months. In the group B of children treated with *Saccharomyces boulardii *the 12-month period of therapy caused a clinical, endoscopic, and microscopic, statistically significant improvement (Figure 5).

In the group C of children with microscopic colitis with presence of foamy macrophages, no therapy was applied.

**Figure 6:**
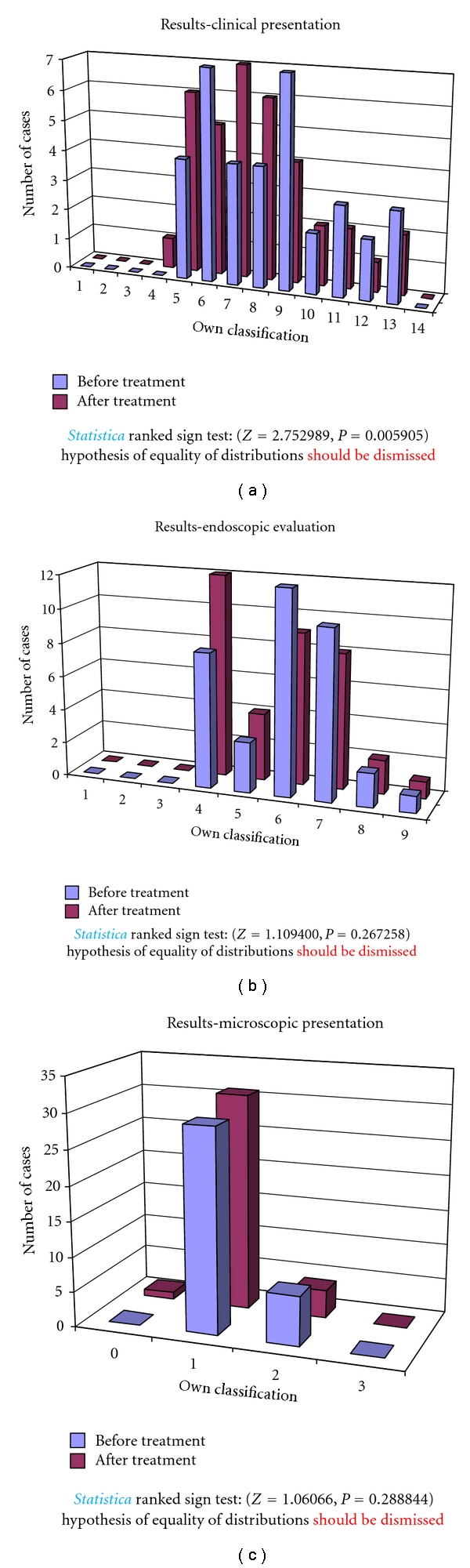
Clinical, endoscopic, and microscopic evaluation of untreated children (group C).

In the group C of untreated children the clinical evaluation showed statistically significant improvement, and the endoscopic and microscopic evaluation showed no regression of pathological lesions (Figure 6).

**Figure 7:**
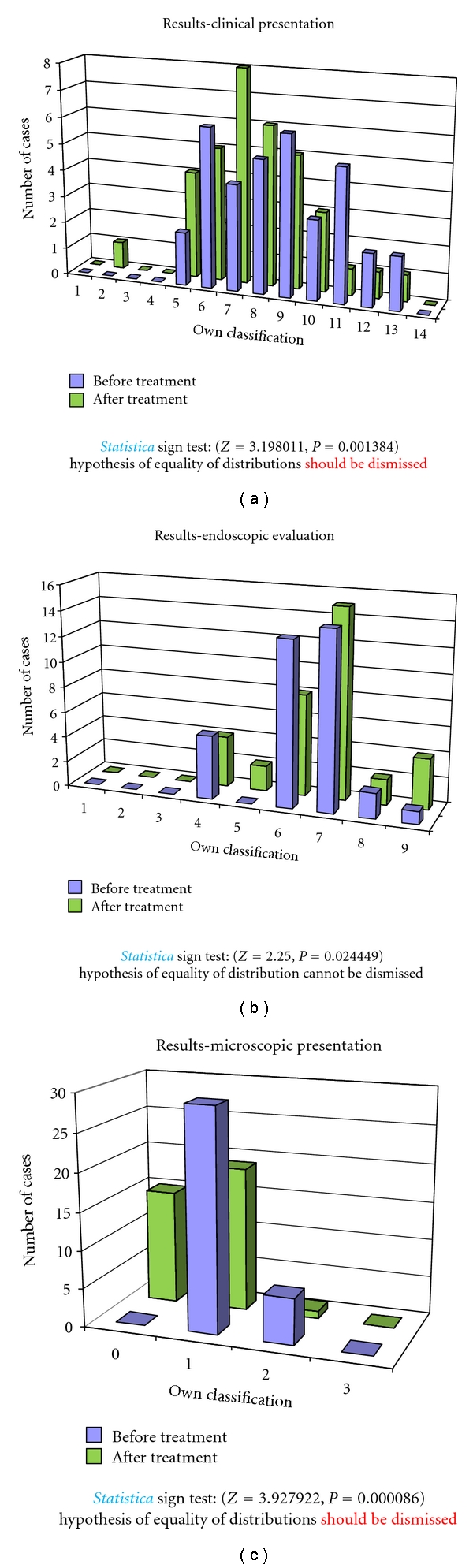
Clinical, endoscopic, and microscopic evaluation of children treated with magnesium (group D).

Children in the group D had magnesium administered for 12 months. Statistical analysis of 12-month magnesium therapy shows microscopic and clinical improvement, but without any endoscopic improvement. Microscopic diagnosis of that unspecific inflammatory disease is relatively easy (Figure 7).

Presence of characteristic foamy macrophages observed at ×200 and ×320 magnification in hematoxylin and eosin-stained specimens is a crucial diagnostic factor [5, 6]. The disease is not associated with destruction of glands or their restructure. The *lamina propria* is not destroyed by lymphocytic infiltration, characteristic for inflammations belonging to the group of IBD. Presence of foamy macrophages in examined histopathological specimens excludes diagnosis of Colitis ulcerosa or Crohn's disease.

In IBD-type inflammations, proliferation of the mononuclear-phagocytic line is characterised by a positive expression of TNF-alpha and IL-beta, absent in our examinations. Microscopic evaluation of properly collected and prepared specimens, but observed under a routinely used low magnification, is a reason for underdiagnosis of the disease in other centres. Observation of the whole group of children confirms previous findings on mild and moderate course of that type of inflammation [4, 6].

Children left without any therapeutic intervention show no endoscopic or microscopic progression of lesions, and an observed surprising clinical improvement indicates an ability for independent regeneration of the intestine in that disease.

Existing reports indicate efficacy of therapy of mild colitis with probiotics. Successful attempts were made in therapy of ulcerative colitis and even of Crohn's disease [8–12]. Part of favourable therapeutic effects of monotherapy with probiotics could be a result of erroneous qualification of microscopic colitis to a group of mild IBD. It seems that probiotics may be useful as supplementary therapy, but their use as a monotherapy is at least risky, particularly in the Crohn's disease.

A favourable clinical effect of *Saccharomyces boulardii* demonstrated in this trial, and confirmed in control endoscopic and microscopic examinations, may be a result of a trophic effect of carbohydrates, B group vitamins (*B_1_, B_2_, B_6_, B_12_*), folic acid, and pantoteic acid contained in that probiotic on enterocytes [8].

Nicotinamide included in *Saccharomyces boulardii *may reduce free oxygen radicals causing intensification of the inflammatory process, and numerous enzymes contained there; for example, proteases and disaccharidases may cause digestion of protein content of foamy macrophages and be responsible for reduction of their count, demonstrated in control microscopic examinations [8, 13].

A favourable therapeutic effect of *Saccharomyces boulardii *may be also a result of its confirmed immunostimulatory and anti-inflammatory effect, significant for limitation and reduction of the inflammatory process in the intestine [13–15].

No favourable therapeutic effect of *5-amino-2-dihydroxybenzoic acid* and relatively mild course of the disease justify resignation from therapy with Mesalazine (*5-amino-2-dihydroxybenzoic acid*) in case of that type of colitis.

A favourable therapeutic effect of magnesium, manifested by regression of microscopic lesions, is most probably a result of supplementation of magnesium deficiency, resulting from erosion of soil and low content of the element in food, and generally low level of supplementation. Besides, mucosa defects found in colitis intensify magnesium deficiency through increased apoptosis.

A favourable effect of magnesium treatment should be probably associated with activating effect of the element on enzymes necessary for synthesis and utilisation of high-energy compounds inside enterocytes. Further observation seems important, expecting development of endoscopic improvement. If it occurs, the microscopic improvement should be treated as a herald of the endoscopic improvement.

Systematic general paediatric and gastroenterological control, along with monitoring of number of foamy macrophages in microscopic examination, plays a very important role in diagnosis and therapy of that type of colitis [2]. The suggested own scale for evaluation of microscopic changes based on quantity of foamy macrophages in the *lamina propia* shows a complete correlation with the clinical and endoscopic evaluation, when analysing the therapeutic effect of *Saccharomyces boulardii* and *5-amino-2-dihydroxybenzoic acid *and a correlation with the endoscopic presentation in case of no therapeutic intervention.

Prospective clinical observation, periodical endoscopic examinations of patients with that disease, will allow verification of currently available knowledge, and possibly will open new diagnostic and therapeutic horizons. Difficulties associated with unanimous classification of a significant part of colitis to a strictly defined group are a serious medical problem [5, 16]. Classification of chronic colitis inadequate to actual condition may negatively influence therapy and prognosis in this large, socially important group of civilisation diseases.

## 4. Conclusions

1. Presence of foamy macrophages within the lamina propria of the large intestine is a deciding factor in diagnosis of that form of microscopic colitis.
2. Identification of foamy macrophages is possible with routine hematoxylin and eosin staining and microscopic evaluation of histological specimens at ×200 or ×320 magnification.
3. Efficacy of *Saccharomyces boulardii *in therapy of microscopic colitis with presence of foamy macrophages was demonstrated.
4. No favourable therapeutic effect was achieved following use of *5-amino-2-dihydroxybenzoic acid* (ASA-5) in case of the disease.
5. The disease shows a tendency for spontaneous remission, but only in terms of clinical presentation.
6. Microscopic and clinical improvement found in case of magnesium therapy may be a herald of expected endoscopic improvement.
7. Further paediatric and gastroenterological followup of children with that type of microscopic colitis is necessary.

## Figures and Tables

**Table 1:** Expression of immunocytochemical markers in the microscopic colitis with presence of foamy macrophages.

Macrophages foamy	Marker
Negative	CD_3_ (Cell T)
Negative	CD_4_ (Cell T)
Negative	CD_22_ (Cell B)
Negative	CD_34_(Cell precursor)
Negative	CD_31_ (Entothelial)
*Positive*	CD_68_ (Macrophages)
Negative	Ki_67_ mitotic activity
Negative	Chromogranin
Negative	Synaptophysin
Negative	Neurofilament
Negative	Actin
Negative	P_53_ mutations
Negative	GFAP glial marker
Negative	TNF alpha
*Positive*	PAS
*Positive*	Osmium tetraoxide
Negative	IL_2_
Negative	Mucicarmin
